# Colorectal Cancer Biomarkers: The Expanding Landscape

**DOI:** 10.3390/cells15211979

**Published:** 2026-10-30

**Authors:** Katarzyna Herbetko, Aleksandra Herbetko, Sara Suwała, Jakub Czarny, Julia Rudno-Rudzińska, Olga Milbrandt, Julita Kulbacka

**Affiliations:** 1 Faculty of Medicine, Wroclaw Medical University, 1 Ludwik Pasteur, 50-367 Wroclaw, Poland; katarzyna.rakoczy@student.umw.edu.pl (K.H.); sara.suwala@student.umw.edu.pl (S.S.); 2 Independent Researcher, 01-850 Warsaw, Poland; olaherbetko@gmail.com; 3 Student Scientific Society, Poznań University of Medical Sciences, 61-701 Poznań, Poland; jakub.czarny14@gmail.com; 4 2nd Department of General and Oncological Surgery, Wroclaw Medical University, Borowska 213, 50-529 Wroclaw, Poland; julia.rudno-rudzinska@umw.edu.pl; 5 Oncology Clinic, Institute of Oncology, Poznań University of Medical Sciences, Szamarzewskiego 84, 60-514 Poznań, Poland; omilbrandt@ump.edu.pl; 6 Department of Molecular and Cellular Biology, Faculty of Pharmacy, Wroclaw Medical University, Borowska 211A, 50-556 Wroclaw, Poland; 7 Department of Immunology and Bioelectrochemistry, State Research Institute Centre for Innovative Medicine, LT-08406 Vilnius, Lithuania

**Keywords:** colorectal cancer, biomarkers, liquid biopsy, circulating tumor DNA, microsatellite instability

## Abstract

Colorectal cancer (CRC) remains one of the most common malignancies worldwide and a leading cause of cancer-related mortality. Recent advances in CRC molecular biology have focused on biomarkers for early detection, prognostic stratification, treatment selection, and disease monitoring. Clinically validated biomarkers, including microsatellite instability (MSI), RAS and BRAF mutations, HER2 amplification, and carcinoembryonic antigen (CEA), guide targeted therapy and predict treatment response. However, tumor heterogeneity, clonal evolution, and microenvironmental influences limit the predictive value of single biomarkers. Recent molecular diagnostics therefore increasingly focus on dynamic and minimally invasive approaches. Circulating tumor DNA (ctDNA) is a promising biomarker for detecting minimal residual disease, monitoring recurrence, and assessing tumor evolution in real time. Multi-omics profiling, spatial transcriptomics, and artificial intelligence further expand tumor characterization except for conventional genomic alterations. Our review integrates common CRC biomarkers with developing liquid biopsy, multi-omics, spatial transcriptomics, and artificial intelligence approaches across early detection, prognosis, treatment selection, and longitudinal monitoring. Future biomarker strategies will likely rely on integrated molecular models combined with longitudinal liquid-biopsy assessment. However, most new biomarkers remain at the investigational stage, and assay heterogeneity, limited standardization, and insufficient prospective clinical validation currently restrict their routine clinical use.

## 1. Introduction

**Figure 1:**
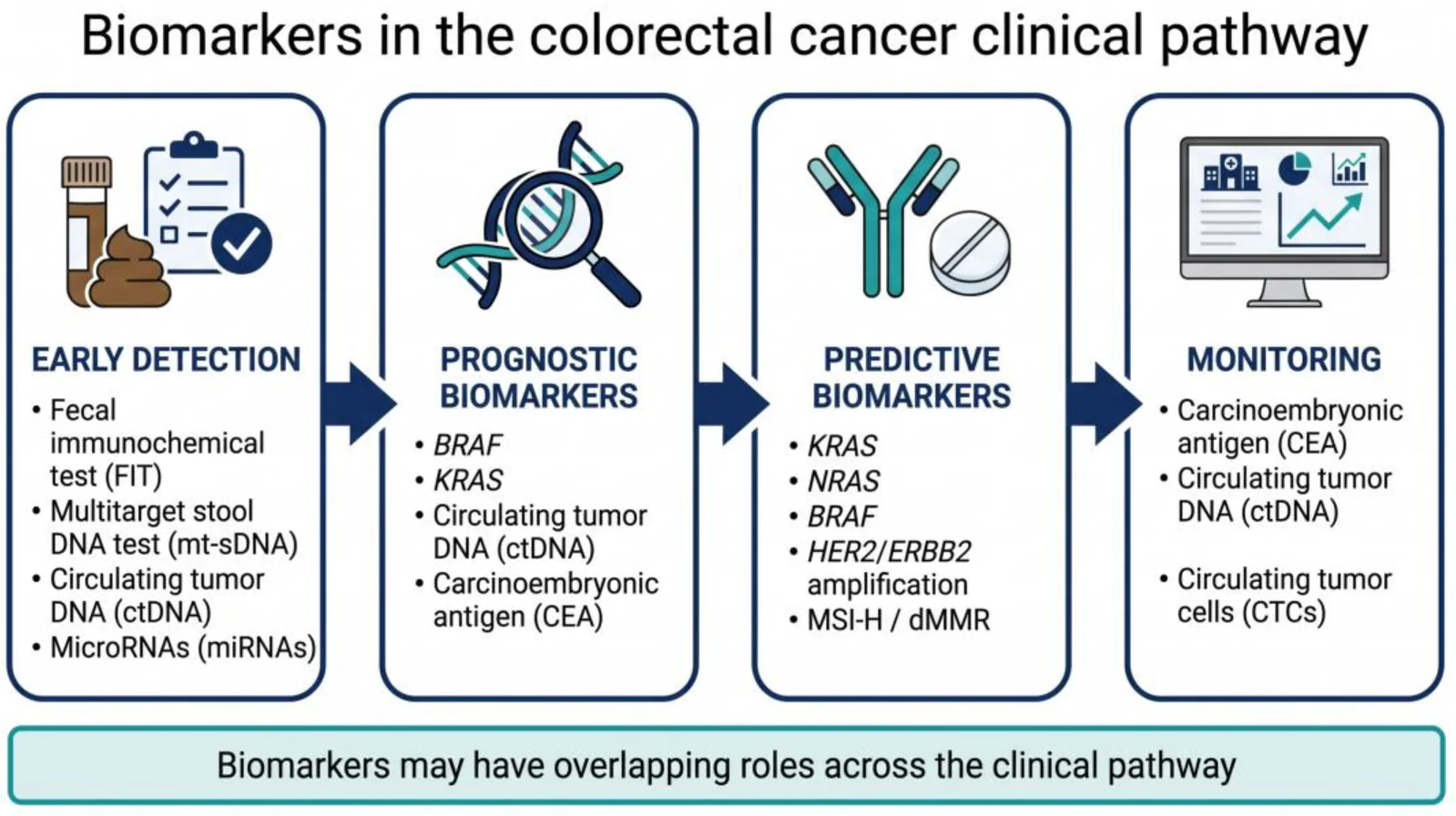
Biomarkers in the colorectal cancer (CRC) clinical pathway. Biomarkers may have overlapping diagnostic, prognostic, predictive, and monitoring roles depending on the clinical context [5,6,7,8,9,10,11,12,13,14,15]. Abbreviations: dMMR, deficient mismatch repair; HER2, human epidermal growth factor receptor 2; MSI-H, microsatellite instability-high. Created in BioRender. Kulbacka, J. (2026) https://BioRender.com/wuj7ja7.

Biomarkers are widely known as measurable indicators of biological or pathological processes that can mirror the presence and progression of the disease as well as the response to therapeutic interventions. Depending on the clinical context they reflect, biomarkers can be classified as diagnostic, prognostic, or predictive. According to the FDA–NIH Biomarker Working Group, a biomarker constitutes “a defined characteristic that is measured as an indicator of normal biological processes, pathogenic processes, or responses to an exposure or intervention” [1,2]. In oncology, biomarkers play a significant role in early detection, prognostic stratification, and prediction of therapeutic response, laying the foundation for precision medicine. When it comes to their role in colorectal cancer (CRC), the third most common type of cancer in the world that is also infamous for being the second leading cause of cancer-related mortality, the identification and clinical implementation of reliable biomarkers have become crucial for disease management and therapeutic decision-making (Figure 1) [3,4].

CRC represents a major global health burden, with more than 1.9 million new cases and nearly 900,000 deaths reported worldwide in 2022 [4]. By 2040, the burden of CRC is predicted to increase with 3.2 million new cases and 1.6 million deaths [16]. Disturbingly, the incidence of CRC diagnosed before the age of 50, referred to as early-onset CRC (EOCRC), has been increasing worldwide in recent years and is often associated with more advanced disease at diagnosis and poorer survival outcomes. From a biomarker perspective, this trend underscores the need to characterize EOCRC’s distinct molecular features and identify biomarkers for earlier detection, risk stratification, and treatment selection [17,18]. The incidence and mortality of CRC vary considerably across geographic regions, with the highest rates observed in developed countries, although increasing trends have recently been reported in many low- and middle-income regions. These differences largely reflect variations in lifestyle, environmental exposures, and access to screening programs. Established risk factors include aging, obesity, insufficient physical activity, smoking, alcohol consumption, and diets rich in processed or red meat. Although highly penetrant hereditary syndromes, including Lynch syndrome (LS) and familial adenomatous polyposis (FAP), account for approximately 5% of CRC cases, familial aggregation studies indicate that inherited factors contribute more broadly to CRC susceptibility. In the Nordic Twin Study of Cancer, genetic factors were estimated to account for 40% (95% CI, 33–48%) of the variation in CRC susceptibility, although estimates were lower for colon and rectal cancer considered separately. A substantial proportion of familial CRC risk therefore remains unexplained by currently recognized Mendelian syndromes and may reflect the combined effects of polygenic risk, rare variants, and shared environmental factors [19,20]. Five-year survival among patients with stage I CRC is 91%, but it declines to approximately 15% in stage IV disease [21]. Thus, early diagnosis is one of the most important determinants of prognosis [22]. Difficulty arises from the fact that the vast majority of patients are diagnosed with CRC once they become symptomatic, and the presence of symptoms, such as rectal bleeding, anemia, and change in the rhythm of bowel movements, is most commonly an indicator of late-stage disease [23,24]. Thus, early diagnosis in asymptomatic patients is one of the main ways to achieve favorable survival outcomes. Although colonoscopy remains the gold standard for CRC detection, patient reluctance and financial constraints limit its use; fecal immunochemical testing (FIT), in turn, is limited by imperfect sensitivity and specificity, resulting in false-positive and false-negative findings [13,25]. The sensitivity of FIT for detecting CRC, using a 100 ng/mL cutoff, reaches 73.8%, compared with 92.3% for a stool-based DNA assay assessing KRAS mutations and aberrant NDRG4 and BMP3 methylation [12,13]. Regarding advanced precancerous lesions, the sensitivity of the discussed tests reaches 28.8% and 42.4% [12], which emphasizes the limitations of the current screening strategy as well as the unmet need for non-invasive, reliable biomarkers. Emerging research addresses this problem and identifies promising markers with diagnostic accuracy, such as circulating tumor DNA (ctDNA) and microRNAs, suggesting that panels combining multiple biomarkers could significantly improve early CRC detection [14,26]. Apart from their usefulness in diagnostics, biomarkers also play a crucial role in both predicting the course of the disease and guiding therapeutic strategies. Prognostic markers, like mutations of KRAS and BRAF as well as ctDNA, can provide information on the risk of occurrence and overall survival (OS), whereas predictive biomarkers enable the selection of targeted therapies and help optimize treatment efficacy while minimizing toxicity [10,11,22,26]. Therefore, the identification and validation of reliable molecular biomarkers remain essential for developing personalized management strategies for CRC.

Recent reviews have addressed biomarkers in gastrointestinal cancers, CRC diagnosis, or selected applications of liquid biopsy and minimal residual disease assessment [5,9,27,28,29]. However, an updated CRC-specific framework integrating established tissue- and blood-based biomarkers with emerging liquid biopsy, multi-omics, spatial transcriptomics, and artificial intelligence approaches remains lacking. The added value of the present review lies in linking individual biomarkers to their principal clinical roles in early detection, prognostic assessment, treatment selection, and longitudinal disease monitoring, while also discussing their current level of clinical validation and the barriers to clinical implementation, including assay standardization, tumor heterogeneity, and the need for prospective validation.

### Review Methodology

This narrative review was based on a structured search of PubMed/MEDLINE, Scopus, and Web of Science Core Collection. The literature search prioritized studies published during the preceding 10 years. However, we included selected older publications when they provided essential historical or methodological foundations, reported landmark biomarker discoveries, described pivotal clinical trials, or established diagnostic and prognostic principles that remain clinically relevant. The search strategy combined terms related to colorectal cancer (“colorectal cancer,” “colon cancer,” “rectal cancer”) with terms related to biomarkers and their clinical applications, including “biomarker,” “molecular marker,” “liquid biopsy,” “circulating tumor DNA,” “minimal residual disease,” “microsatellite instability,” “mismatch repair,” “KRAS,” “NRAS,” “BRAF,” “HER2,” “CEA,” “microRNA,” “DNA methylation,” “multi-omics,” “spatial transcriptomics,” and “artificial intelligence.” The reference list of key publications was also screened to identify relevant milestone studies and current guidelines. We included English-language human studies, clinical trials, observational studies, systematic reviews, narrative reviews, meta-analyses, and clinical guidelines or consensus statements addressing CRC biomarkers or their clinical implementation. Publications unrelated to CRC biomarkers, duplicate records, conference abstracts without sufficient data, and studies lacking relevant clinical or translational information were excluded. Titles and abstracts were screened for relevance, followed by full-text assessment of potentially eligible publications. Because this was a narrative review, publications were selected purposively according to their clinical relevance, methodological quality, and contribution to the objectives of the review.

## 2. Established Clinical Biomarkers

Several molecular biomarkers are already incorporated into routine clinical practice for CRC management. These markers provide valuable information regarding tumor biology, prognosis, and potential response to targeted therapies, thereby supporting personalized treatment strategies. Current clinical guidelines recommend assessing specific genomic alterations and molecular characteristics to guide therapeutic decisions, particularly in advanced disease. The most widely recognized and clinically validated biomarkers in CRC include microsatellite instability (MSI), mutations in the RAS and BRAF genes, HER2 amplification, and serum carcinoembryonic antigen (CEA) levels [10,15,22].

### 2.1. MSI/Deficient Mismatch Repair (dMMR)

**Figure 2:**
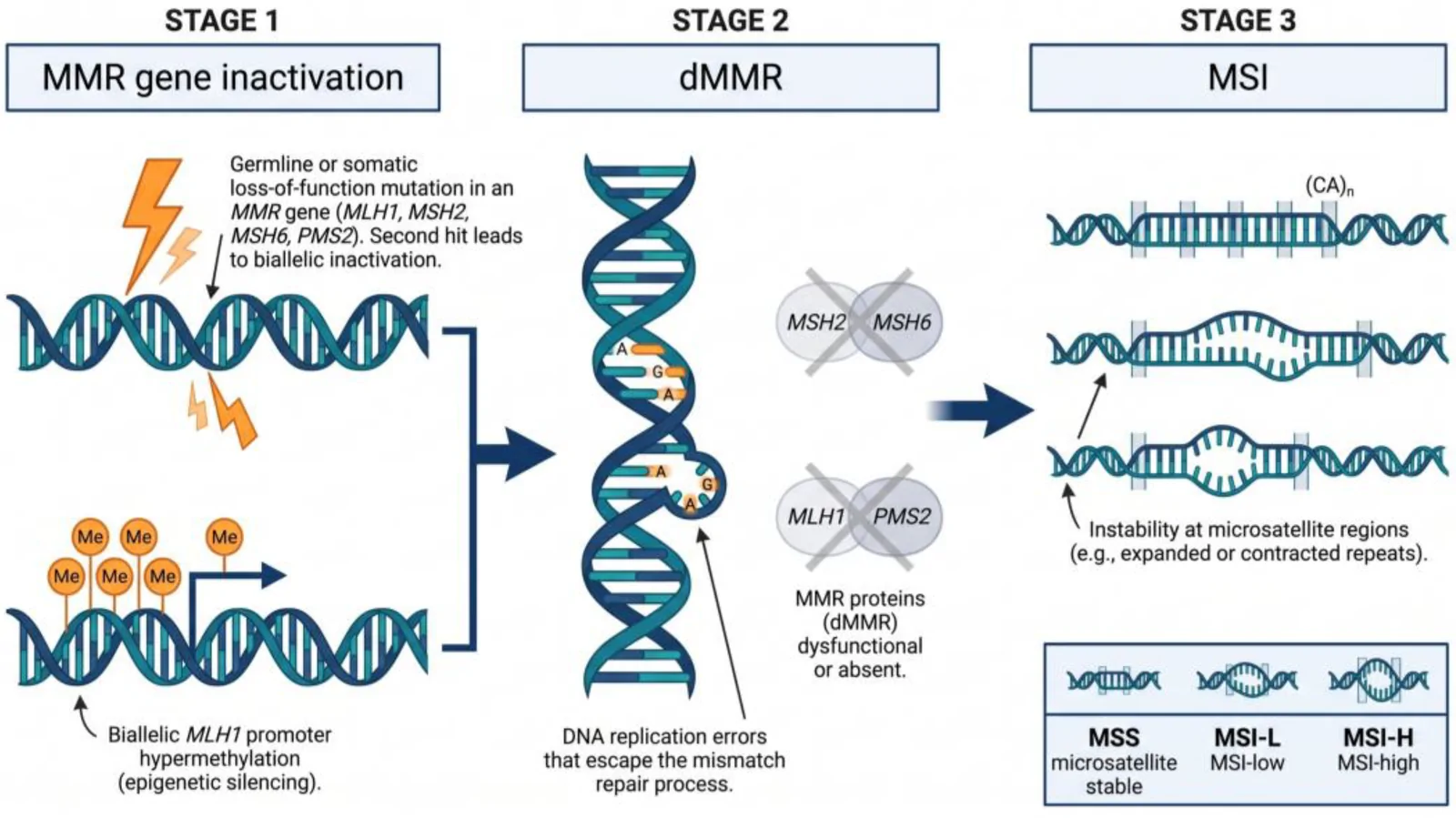
Mechanisms of deficient mismatch repair (dMMR) and microsatellite instability (MSI). Symbols: crosses (X) over the MSH2–MSH6 and MLH1–PMS2 heterodimers indicate that these MMR protein complexes are dysfunctional or absent (dMMR). Abbreviations: MMR, mismatch repair; dMMR, deficient mismatch repair; MSI, microsatellite instability [30,31,32]. Created in BioRender. Kulbacka, J. (2026) https://BioRender.com/p54lwcb.

DNA mismatch repair (MMR) and MSI status are invaluable biomarkers that guide clinical management of CRC patients [30]. MMR proteins recognize and repair base–base mismatches and insertion–deletion loops generated during DNA replication, thereby maintaining genomic stability. In humans, mismatch recognition is mediated by two MutS heterodimers: MutSα (MSH2–MSH6), which preferentially recognizes base–base mismatches and small insertion–deletion loops, and MutSβ (MSH2–MSH3), which preferentially recognizes larger insertion–deletion loops. Following mismatch recognition, MutLα (MLH1–PMS2) is recruited to the MutS–DNA complex and coordinates downstream repair. Its PMS2 subunit provides mismatch-activated endonuclease activity that introduces nicks into the newly synthesized strand, thereby enabling excision of the error-containing strand and subsequent DNA resynthesis [31]. Alterations in genes involved in the MMR pathway are frequently observed in sporadic CRC. Moreover, germline mutations in MMR genes are associated with LS, the most common hereditary form of CRC. dMMR constitutes a consequence of the inactivation of one or more core MMR genes, which may occur through germline variants, as observed in LS, or through somatic loss-of-function mutations followed by a second hit leading to biallelic gene inactivation. In addition, MMR deficiency may arise from epigenetic silencing caused by MLH1 promoter hypermethylation. The loss of functional MMR proteins leads to the accumulation of replication errors in microsatellite regions, resulting in MSI. Depending on the extent of instability detected across microsatellite loci, MSI can be classified as microsatellite stable (MSS), MSI-low (MSI-L), and MSI-high (MSI-H) (Figure 2) [31,32]. MMR/MSI status is used not only to identify LS, but also to predict immune checkpoint inhibitors (ICIs) treatment response [33]. MSI/dMMR status can be assessed by immunohistochemistry for MLH1, PMS2, MSH2, and MSH6; PCR-based MSI testing; or a validated NGS assay. Universal tumor testing is recommended for newly diagnosed CRC [31]. Loss of MLH1/PMS2 should be followed by BRAF V600E and/or MLH1 promoter methylation testing to exclude a sporadic tumor. Patients with abnormal MSH2, MSH6, or PMS2 expression, or with MLH1/PMS2 loss without a sporadic explanation, should be referred for genetic counseling and germline testing for Lynch syndrome [34].

Clinically, dMMR/MSI-H CRC tumors usually present as poorly differentiated mucinous adenocarcinomas and are more frequently located in the proximal colon [35,36]. In the sporadic setting, BRAF V600E commonly occurs within the serrated neoplasia pathway and is strongly associated with CpG island methylator phenotype-high (CIMP-high) tumors, which are characterized by widespread promoter CpG-island hypermethylation. In a substantial proportion of these cancers, hypermethylation of the MLH1 promoter causes transcriptional silencing of MLH1, resulting in dMMR and the MSI-H phenotype [36,37]. dMMR/MSI-H is more common in early-stage CRC, likely because high tumor mutational burden (TMB) generates neoantigens that promote CD8^+^ T-cell infiltration and an immunogenic tumor microenvironment (TME). However, in metastatic disease, dMMR/MSI-H is associated with poorer prognosis, which may partly reflect the higher prevalence of the BRAF V600E mutation rather than the MSI status itself [36,38]. Regarding therapeutic implications, although CRC patients with MSI-H generally respond favorably to ICIs, treatment response depends on specific modifying factors. First, disruptions in the gut microbiota ecosystem caused by broad-spectrum antibiotics can negatively affect the therapeutic potential of ICIs, whereas appropriate modulation of the microbiome may improve immunotherapy outcomes [39]. For instance, although Fusobacterium nucleatum is widely regarded as a pro-tumorigenic and immunosuppressive microorganism in CRC and has been associated with adverse clinical outcomes, some studies suggest it may enhance responses to programmed death-ligand 1 (PD-L1) blockade in specific experimental contexts [40]. Secondly, peritoneal metastases accompanied by ascites noticeably reduce the effectiveness of ICIs, probably because of the combined effects of malignant ascites and immunosuppressive TME [41]. These findings question the use of MSI-H as a standalone predictive biomarker, underscoring the need for a composite biomarker approach.

Neoadjuvant immunotherapy has also produced practice-changing results in localized dMMR/MSI-H CRC. In the NICHE-2 study, neoadjuvant nivolumab plus ipilimumab produced pathological responses in 98% of patients and a pathological complete response in 68%, with no recurrences reported at a median follow-up of 26 months [42]. In locally advanced dMMR rectal cancer, neoadjuvant dostarlimab has also enabled organ-preservation strategies in patients achieving a complete clinical response [43]. Nevertheless, surgery should currently be omitted only in carefully selected patients managed within structured surveillance protocols or clinical trials.

In contrast to MSI-H CRC, MSS CRC, in which microsatellite length and composition remain stable during replication, is typically associated with proficient mismatch repair (pMMR). MSS/pMMR tumors are characterized by low TMB and limited infiltration of immune cells within the TME, which results in poor response to ICI monotherapy [44]. Importantly, MSS CRC accounts for approximately 85–90% of colorectal tumors. It is usually located in the left colon and rectum and presents as well-differentiated or moderately differentiated adenocarcinomas. The immune landscape of MSS tumors is generally dominated by immunosuppressive cell populations, including regulatory T cells and M2 macrophages, and is characterized by relatively low PD-L1 expression, which collectively facilitates immune evasion [45].

From a prognostic perspective, outcomes in MSS CRC are strongly influenced by tumor stage and the characteristics of the TME. While survival outcomes in early-stage MSS disease may be comparable to MSI-H tumors, patients with advanced MSS CRC often demonstrate significantly poorer survival, likely due to both chemotherapy resistance and an immunosuppressive TME. Interestingly, recent studies highlight the prognostic importance of tumor-infiltrating lymphocyte (TIL) density in MSS CRC. Even within the MSS group, patients with high TIL infiltration (MSS-TIL-H) have shown significantly improved overall and disease-free survival (DFS) compared with those with low TIL levels, indicating that immune contexture remains an important determinant of prognosis [46]. Given the limited efficacy of immunotherapy in MSS CRC, current research focuses on combination therapies, such as combining vascular endothelial growth factor receptor (VEGFR) inhibitors with PD-1 blockade and dual immune checkpoint inhibition, which have shown encouraging activity in refractory MSS metastatic CRC (mCRC). In parallel, increasing attention is being directed toward biomarker-driven therapeutic strategies, including the identification of predictive markers such as TIL density, POLE/POLD mutations, and TMB, which may help identify subgroups of MSS CRC patients more likely to benefit from immunotherapeutic approaches [46,47,48]. Recent advances in digital pathology and artificial intelligence (AI) have also improved MMR/MSI status evaluation in CRC. Several studies have shown that deep learning (DL) algorithms applied to digitized whole-slide images can accurately predict dMMR/MSI-H status, offering a rapid, scalable approach to tumor classification [49,50]. In addition to MSI detection, DL-based models can extract clinically relevant prognostic and predictive features from routine hematoxylin and eosin (H&E)-stained slides, enabling the development of computational prognostic models that may outperform traditional morphological and molecular markers [51]. These tools have shown consistent performance across tumor and nodal stages and may support personalized treatment decisions, including identifying patients who may benefit from intensified therapy or, conversely, those who may avoid overtreatment [52]. Moreover, machine learning (ML) approaches integrating clinical, pathological, and transcriptomic data are increasingly being explored to predict immunotherapy responses in CRC, highlighting the growing potential of AI-assisted diagnostics in precision oncology [53,54].

MSI/dMMR status is an established biomarker of sensitivity to immune-checkpoint inhibitors. In clinical practice, MSI/dMMR testing identifies patients who may benefit from pembrolizumab or nivolumab-based immunotherapy in advanced CRC [55,56]. Neoadjuvant immunotherapy may also support organ-preservation strategies in carefully selected patients with localized dMMR/MSI-H disease [42,43]. POLE/POLD1 alterations may identify additional hypermutated tumors with potential sensitivity to immune-checkpoint inhibitors, but the available evidence remains limited, and these alterations should not be considered equivalent to MSI-H/dMMR [57]. Other immune biomarkers, including PD-1/Tim-3 coexpression, HLA class I expression, tumor-infiltrating lymphocytes, MCL-1, and CCR5, have been associated with prognosis or immunotherapy response in exploratory cohorts. However, these markers lack standardized assays, validated thresholds, and prospective evidence showing that biomarker-guided selection improves outcomes and should therefore remain investigational [46,58,59,60,61].

### 2.2. RAS Mutations

**Figure 3:**
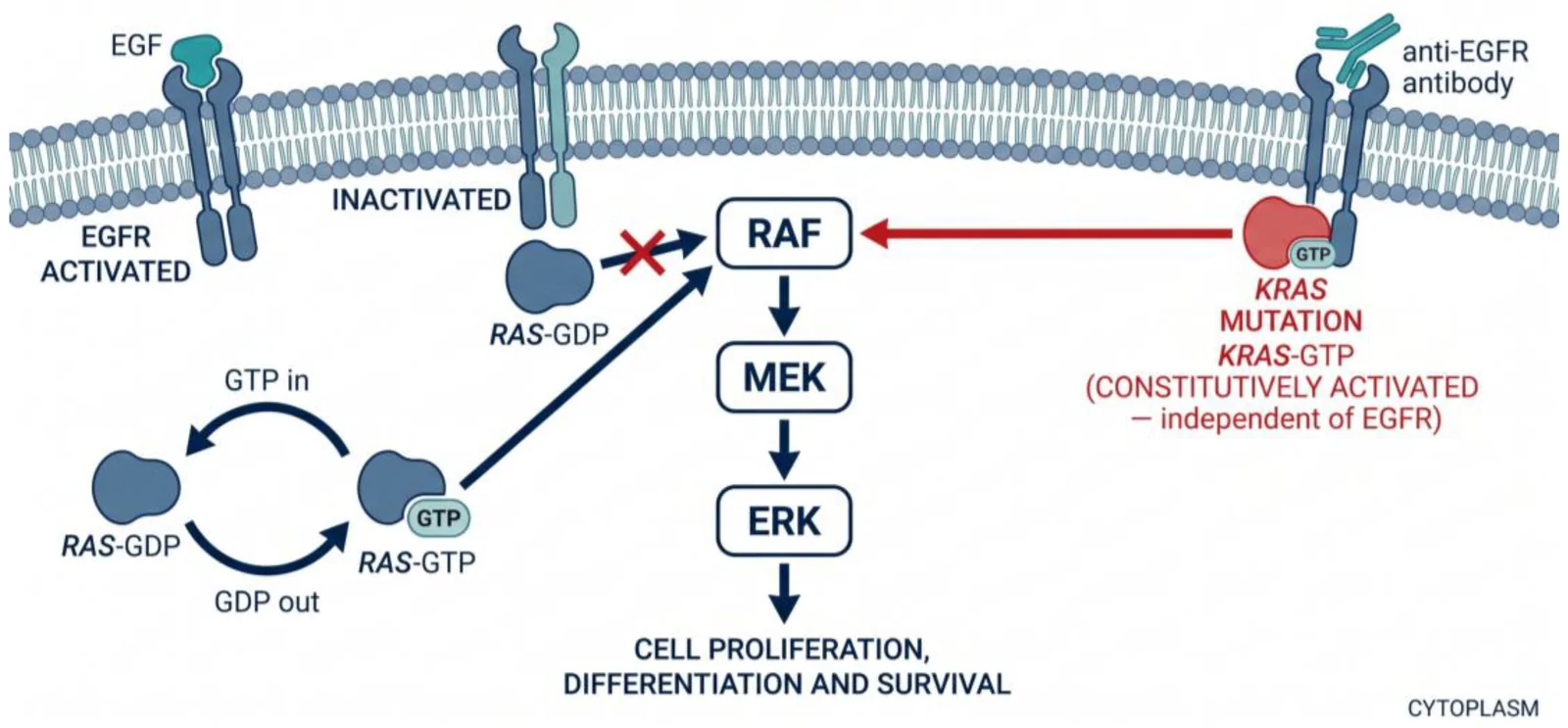
EGFR–RAS–RAF–MEK–ERK (MAPK) signaling in colorectal cancer and the effect of KRAS mutation. With ligand-bound EGFR, RAS cycles between an inactive GDP-bound and an active GTP-bound state, and RAS-GTP activates RAF, which sequentially phosphorylates MEK and ERK. Without ligand, RAS remains GDP-bound and signaling stops. Mutant KRAS is locked in the GTP-bound state and activates RAF constitutively, independently of upstream EGFR, so anti-EGFR antibodies fail to switch off the cascade [62,66,78,79]. Symbols: the red cross (X) indicates that inactive, GDP-bound RAS does not activate RAF; the red arrow indicates constitutive, EGFR-independent activation of RAF by mutant KRAS-GTP, which persists despite EGFR blockade by anti-EGFR antibodies. Created in BioRender. Kulbacka, J. (2026) https://BioRender.com/bpevgui.

Mutations in the RAS superfamily have been validated as drivers of tumor initiation and survival in numerous studies [62]. RAS proteins function as GTPase molecular switches that alternate between inactive GDP-bound and active GTP-bound states. In their active conformation, they interact with downstream effector proteins to initiate signaling pathways. In CRC, KRAS mutations occur in approximately 40–45% of tumors, whereas NRAS mutations are much less frequent, affecting about 3–5% of cases, and HRAS mutations are exceedingly rare [63]. KRAS mutations, which usually involve alterations in codons 12 and 13, with the most common variants including G12D, G12V, and G13D, are associated with poor tumor differentiation, distant metastases, and shorter recurrence-free survival (RFS) and OS [63,64,65,66]. Nevertheless, the prognostic significance of KRAS mutations may vary depending on the specific mutation subtype and disease stage. For instance, while codon 12 mutations are often associated with poorer overall survival and DFS, the prognostic impact of codon 13 alterations remains less clear. Furthermore, in stage II–III CRC, KRAS mutation status has not consistently demonstrated significant prognostic relevance [67]. Heterogeneity in KRAS mutation status between primary tumors and distant metastases is generally uncommon, as it occurs in less than 10% of cases [68,69]. However, pulmonary metastases demonstrate substantially higher discordance rates, reaching up to 30% in comparison with other metastatic sites [70,71]. This finding supports testing metastatic tissue whenever feasible, particularly in pulmonary metastases, and repeating RAS testing when progression occurs or when molecular results from different disease sites are discordant. Moreover, both wild-type-to-mutant and mutant-to-wild-type mutations have been reported in metastatic lesions [72]. Discussed findings suggest that KRAS-mutant subclones may be present in both primary and metastatic tumors, potentially contributing to therapeutic response [73]. Analyses of ctDNA have demonstrated that previously undetected KRAS mutations can emerge during therapy, reflecting the expansion of resistant tumor clones [74]. Conversely, some patients initially classified as RAS-mutant may exhibit loss of detectable mutations in plasma during disease progression, suggesting potential therapeutic implications for liquid biopsy–based reassessment of RAS status during treatment [75,76]. From a molecular perspective, KRAS consists of two principal structural components: the G-domain and the C-terminal hypervariable region. The G-domain includes the switch I, switch II, and P-loop regions, which undergo conformational changes depending on whether GDP or GTP is bound [65,77]. Under physiological conditions, epidermal growth factor receptor (EGFR) activation leads to RAS activation via GTP binding, which then triggers downstream signaling through the MAPK pathway, a key regulator of cell proliferation, differentiation, and survival [66,78]. Once a KRAS mutation occurs, the protein remains constitutively bound to GTP, persistently activating this signaling cascade independent of upstream EGFR stimulation (Figure 3). This promotes uncontrolled tumor growth.

RAS status is therefore a core predictive biomarker for anti-EGFR therapy. Activating KRAS or NRAS mutations indicate primary resistance to cetuximab and panitumumab, whereas RAS wild-type status identifies patients who may be eligible for anti-EGFR treatment [11,15]. The dedicated Section ”Anti-EGFR Therapy: Predictive Biomarkers and Resistance Mechanisms” on mechanisms of resistance discusses acquired resistance mechanisms, including emergent RAS or BRAF alterations, HER2 activation, PIK3CA/PTEN dysregulation, and EGFR extracellular-domain mutations [75,80,81]. In previously treated patients with KRAS G12C-mutated mCRC, targeted combinations such as adagrasib plus cetuximab or sotorasib plus panitumumab represent additional treatment options [82,83].

### 2.3. BRAF Mutations

BRAF, a serine/threonine kinase, plays a central role in the MAPK signaling cascade, regulating cell proliferation and survival. Activating BRAF mutations occur in about 8–10% of CRC cases and constitutively activate the MAPK pathway, promoting tumor growth and progression [84]. The vast majority of BRAF alterations are the V600E mutations, which account for about 90–95% of BRAF-mutant CRC cases and exhibit markedly increased kinase activity compared with wild-type BRAF [85]. Clinically, BRAF V600E mutations are associated with rapid disease progression and poor prognosis, especially in mCRC. They usually occur in the right colon, show mucinous histology, and are more common in older patients and women [84]. Regarding interactions with other biomarkers, BRAF mutations are detected in a substantial proportion of sporadic MSI-H CRC, particularly those with MLH1 promoter hypermethylation [86]. MSI-H status might partially mitigate the otherwise adverse prognostic impact of BRAF mutations. In contrast, MSS BRAF-mutant tumors exhibit particularly poor survival outcomes [84,86,87]. BRAF mutations are divided into functional classes: class I (V600), class II (RAS-independent non-V600), and class III (RAS-dependent). Class II mutations are typically resistant to anti-EGFR therapy, whereas class III mutations may retain sensitivity to EGFR inhibitors [88]. BRAF V600E can also be assessed with the VE1 mutation-specific immunohistochemical assay, which provides rapid, tissue-based screening. However, its diagnostic performance in CRC depends on the staining platform, antibody interpretation, tumor heterogeneity, and positivity threshold. Therefore, molecular testing should confirm confusing or discordant results. VE1 detects the V600E protein and does not identify non-V600E BRAF variants [89]. Importantly, targeted therapeutic strategies have emerged for CRC with BRAF V600E mutations. Combination therapy with the BRAF inhibitor encorafenib and the anti-EGFR antibody cetuximab has demonstrated improved OS, progression-free survival (PFS), and response rates in previously treated mCRC patients [90]. In addition to targeted therapy, BRAF mutation status also influences response to immunotherapy. Clinical trials such as KEYNOTE-177 and CheckMate-142 have demonstrated that ICIs, including pembrolizumab and nivolumab (alone or combined with ipilimumab), provide significant clinical benefit in MSI-H mCRC, including cases harboring BRAF mutations [55,56]. Consequently, BRAF mutation status represents both an important prognostic biomarker and a predictive marker for targeted and immunotherapeutic strategies in CRC.

### 2.4. HER-2 Amplification

ERBB2, also known as CD340 and HER-2, is a protein tyrosine kinase receptor, the locus of which is localized on chromosome 17q12 [91]. HER-2 alterations, including gene amplification and activating point mutations, are detected in a small subset of CRCs. Gene amplification occurs in about 2–5% of cases and is more frequent in RAS wild-type tumors, whereas the association with higher tumor mutational burden appears to depend on the specific ERBB2 alteration and molecular context [92]. HER2 overexpression is more frequently detected in tumors of the left colon and rectum [93]. Within the complex molecular interactions between HER-2 and other biomarkers, MSI-H was not found in tumors with HER-2 amplification, whereas the correlation between point mutations in HER-2 and MSI-H CRC is positive [94]. The prognostic significance of the discussed biomarker remains controversial. According to some studies, HER-2 amplification is associated with increased invasive potential of the tumor and poorer survival outcomes [95,96,97], whereas others claim that either survival is improved or there is no association at all [98,99]. These differences may reflect anatomical and molecular heterogeneity in CRC, differences in assay platforms (including immunohistochemistry, in situ hybridization, and sequencing), variation in interpretation cut-offs, and differences in study populations and clinical settings. As a predictive biomarker, HER-2 alterations predict resistance to anti-EGFR therapy in CRC. Both HER-2 amplification and heregulin-mediated HER-3 activation sustain ERK1/2 signaling and continued tumor growth. In patients with wild-type KRAS/NRAS/BRAF/PIK3CA, HER2 amplification has been associated with shorter response duration to EGFR inhibitors and poorer prognosis, while emerging data also highlight HER2 as a therapeutic target for HER2-directed agents such as antibody–drug conjugates (ADCs) [100]. Currently, ADCs, monoclonal antibodies, and tyrosine kinase inhibitors (TKIs), with combination regimens such as trastuzumab–pertuzumab or trastuzumab-based therapies, are under investigation and show promising activity in HER2-positive, RAS wild-type mCRC [100,101,102]. Most importantly, tucatinib plus trastuzumab is the only CRC-specific FDA-approved HER2-directed regimen for RAS wild-type, HER2-positive unresectable or metastatic CRC that has progressed after fluoropyrimidine-, oxaliplatin-, and irinotecan-based therapy. This approval was based on the MOUNTAINEER trial [103].

### 2.5. CEA

CEA represents one of the most widely recognized tumor markers across multiple cancer types. However, a high rate of false positives limits its usefulness for CRC diagnosis when assessed without broader molecular context [104]. Nevertheless, multimarker serum panels may improve diagnostic performance compared with single markers. In a study of 1027 samples, a six-marker panel comprising CEA, CYFRA 21-1, ferritin, osteopontin, anti-p53, and seprase achieved 69.6% sensitivity at 95% specificity, compared with 43.9% for CEA alone [105]. Interestingly, recent research suggests that for both early-stage CEA and even the precancerous stage of disease, fecal CEA may be a more sensitive biomarker than serum CEA, offering improved diagnostic precision through a non-invasive approach [106]. Although CEA’s diagnostic potential remains limited, it plays a significant role in detecting CRC metastases. Integrating CEA interpretation with magnetic resonance imaging (MRI) increases the accuracy of liver metastasis diagnosis [107]. Given CEA’s prognostic value, it is widely used to predict the risk of CRC recurrence after surgery. An increase in the initial level of this biomarker is associated with a greater risk of CRC recurrence, whereas an elevation at recurrence is related to lower five-year DFS rates [108,109]. However, serum CEA alone cannot confirm or exclude tumor recurrence and should be interpreted together with clinical assessment and imaging.

### 2.6. Other Clinically Investigated Biomarkers

Researchers have investigated additional biomarkers for potential clinical utility in CRC. Mutations in the TP53 tumor suppressor gene occur in approximately 50–60% of colorectal tumors and are associated with genomic instability and disease progression [110]. Similarly, PIK3CA mutations, which activate the PI3K/AKT signaling pathway, have been implicated in tumor growth and may influence the response to targeted therapies [111]. Protein-based biomarkers such as vascular endothelial growth factor (VEGF) and the proliferation marker Ki-67 have also been extensively studied. VEGF plays a key role in tumor angiogenesis and may predict response to antiangiogenic therapies such as bevacizumab [112], while Ki-67 expression reflects tumor proliferative activity and has been associated with aggressive tumor behavior [113]. None of these markers currently has a routine guideline-supported role in treatment selection. TP53, PIK3CA, VEGF, and Ki-67 should therefore be regarded as exploratory or research-oriented biomarkers, in contrast to established biomarkers such as MSI/dMMR, RAS, BRAF, and HER2, which inform guideline-based treatment decisions in appropriate clinical settings [15,114,115].

## 3. Emerging Biomarkers

Despite the important role of established biomarkers in guiding CRC management, their clinical utility remains limited by their restricted sensitivity, tumor heterogeneity, and inability to fully capture the disease’s dynamic molecular evolution. Advances in molecular technologies have therefore stimulated the search for novel biomarkers that could improve early detection, enable real-time monitoring of tumor burden, and refine prognostic and predictive stratification. Among these emerging approaches, liquid biopsy has attracted considerable attention because it is minimally invasive and can reflect the tumor’s current molecular landscape by analyzing circulating tumor-derived components such as ctDNA, RNA molecules, and circulating tumor cells (CTCs) [6,116,117]. Despite liquid biopsy and multi-omics, genome-wide methylation profiles, circulating miRNAs, telomere length, germline polymorphisms, serum proteins, and chromosomal alterations have been associated with prognosis or treatment response in CRC [118,119]. However, most available evidence comes from retrospective or single-study cohorts, and assay platforms, thresholds, patient populations, and clinical endpoints vary substantially. None of these markers is currently sufficient for routine clinical decision-making or guideline-based treatment selection [120,121,122,123,124,125]. They should therefore be considered investigational. Future studies should use standardized assays, prospective multicenter validation, and predefined clinical endpoints, while demonstrating added value beyond established clinicopathological and molecular factors.

### 3.1. Liquid Biopsy in CRC: Current Evidence and Clinical Scope

#### 3.1.1. Biological Background

Liquid biopsy encompasses several tumor-derived components detectable in blood. ctDNA is released into circulation through apoptosis, necrosis, and active secretion, reflecting the tumor’s current biological state. Its molecular profile retains carcinogenic alterations, including changes in the APC, TP53, and KRAS genes, which are among the most clonal alterations in advanced CRC [80]. This feature allows ctDNA to serve as a dynamic sensor of disease, often providing information before radiological progression. Other circulating molecules, including miRNAs and long non-coding RNAs (lncRNAs), are also being investigated. Serum miR-21 levels have been associated with CRC aggressiveness and decrease after tumor resection, supporting their potential diagnostic and prognostic value [126]. Epigenetic studies further suggest that lncRNAs and histone modifications may contribute to CRC biology and could be incorporated into future liquid-biopsy panels [127]. Nevertheless, ctDNA remains the liquid-biopsy biomarker with the highest level of clinical validation [117].

**Figure 4:**
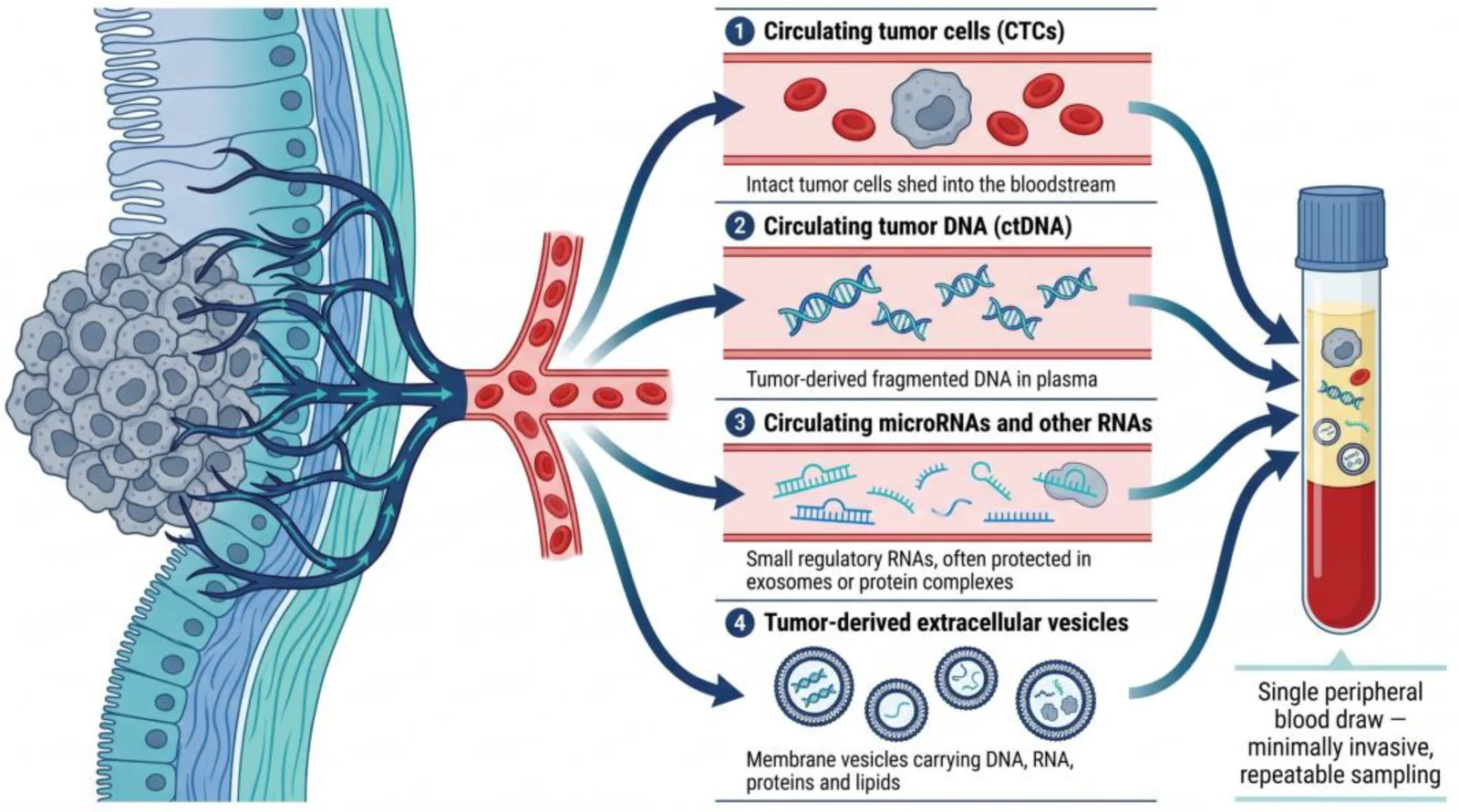
Major liquid-biopsy components in colorectal cancer (CRC). Liquid biopsy enables the analysis of circulating tumor DNA (ctDNA), circulating tumor cells (CTCs), circulating microRNAs and other non-coding RNAs, and tumor-derived extracellular vesicles. These analytes may support early detection, prognostic assessment, treatment monitoring, and minimal residual disease (MRD) evaluation. Abbreviations: ctDNA, circulating tumor DNA; CTCs, circulating tumor cells; MRD, minimal residual disease [6,7,8,26,116,117,133]. Created in BioRender. Kulbacka, J. (2026) https://BioRender.com/93g7mtg.

Other circulating tumor-derived components, including CTCs and extracellular vesicles, may provide complementary information on metastatic potential, tumor heterogeneity, and treatment response (Figure 4) [26,116]. CTCs provide complementary prognostic information. Plastin 3 (PLS3)-positive CTCs are associated with poor prognosis, particularly in patients with stage II–III CRC [128]. Specific CTC features also relate to prognosis: PLS3-positive CTCs are associated with poor outcomes; a six-gene assay (CEA, EpCAM, CK19, MUC1, EGFR, and c-MET) improves disease monitoring and correlates with PFS [129]. High CTC counts and mesenchymal phenotype (M+ CTCs) correlate with metastasis and shorter PFS and OS [130]. Akt-2, ALDH1, or PI3Kα expression in CTCs identifies patients with poorer outcomes [131], while COX-2–positive CTCs may indicate more aggressive disease [132]. Extracellular vesicles, particularly tumor-derived exosomes, contain proteins, DNA fragments, and regulatory RNAs that may influence intercellular communication and serve as non-invasive indicators of tumor activity [133].

#### 3.1.2. ctDNA in Minimal Residual Disease (MRD) Assessment

In patients with localized colon and rectal cancer, the presence of ctDNA after resection of the primary tumor has been shown to be one of the strongest known risk factors for recurrence and death, surpassing clinical-pathological and imaging parameters. Studies by Tie and colleagues have shown that ctDNA-negative patients after stage II surgery have a very low risk of recurrence, while ctDNA-positive patients have a significantly higher risk [134,135]. A meta-analysis by Chidharla et al. confirms that ctDNA after treatment is associated with significantly shorter RFS and OS [136]. Similar observations were made in rectal cancer after neoadjuvant therapy, where ctDNA strongly differentiated prognosis even before surgery, supporting the interpretation of ctDNA as a molecular equivalent of oncological margins. An important extension of this concept is using ctDNA in oligometastatic disease treated with radical intent. In this group, classic prognostic parameters such as the number of metastases, their location, or margin status have proven insufficiently accurate for predicting recurrence. Research by Marmorino and colleagues has shown that detectable ctDNA after resection of liver metastases in CRC is an independent and strong marker of residual disease, predicting both time to recurrence and survival [137]. Similarly, O’Donnell and co-authors observe that ctDNA after completion of locoregional treatment (resections, ablations, stereotactic radiotherapy) enables earlier and more accurate identification of patients at risk of recurrence than traditional clinical variables [138]. The commentary by Yan et al. synthesizes data showing that liquid biopsy not only allows for the detection of disease, but above all, for the dynamic assessment of MRD, risk of recurrence, and response to treatment at various stages of CRC [116]. The prognostic role of post-treatment ctDNA in oligometastatic CRC is discussed in greater detail below.

In clinical practice, these findings support using ctDNA as a dynamic marker of recurrence risk and for treatment monitoring. In the clinical setting, postoperative ctDNA positivity is associated with a higher risk of recurrence, while serial testing may detect molecular relapse before radiological progression [136,139]. Prospective randomized evidence supports ctDNA-guided adjuvant decisions in selected stage II colon cancer settings, but the broader clinical role remains assay-, timing-, and disease-setting dependent [139,140]. ctDNA should therefore complement, rather than replace, clinicopathological assessment and imaging until prospective studies demonstrate that acting on ctDNA results improves patient outcomes [140,141].

#### 3.1.3. Epigenetic Signals in Liquid Biopsy

Liquid-biopsy components have different potential clinical applications and should not be considered interchangeable. Mutational ctDNA analysis is primarily suited to tracing clonal evolution, quantifying tumor burden, and detecting emerging mechanisms of therapeutic resistance, including the re-emergence of RAS-mutant clones during anti-EGFR therapy [117]. Plasma DNA methylation markers reflect recurrent epigenetic changes and may be particularly useful for blood-based screening and broad detection of tumor-derived signals. Methylated SEPT9 is a representative example; however, its sensitivity remains insufficient to replace FIT or colonoscopy, particularly for detecting advanced precancerous lesions [142,143].

Circulating miRNAs and lncRNAs reflect regulatory pathways associated with proliferation, epithelial–mesenchymal transition, invasion, and treatment response. Nevertheless, the current evidence remains predominantly retrospective, single-study, or laboratory-based, and these markers have not been validated for routine clinical decision-making in CRC [126,144].

Integrated plasma assays combining mutation and methylation signals, such as Guardant Reveal, may provide tumor-naïve MRD assessment without prior tumor sequencing. However, their clinical performance remains assay-specific, and results obtained from different MRD platforms should not be considered directly interchangeable [144,145].

#### 3.1.4. Oligometastatic Disease

In a prospective single-center study of 25 patients with oligometastatic CRC treated with definitive local therapy, detectable ctDNA after treatment was associated with worse overall survival (HR 11.28; 95% CI, 1.31–97.05), whereas baseline ctDNA was not significantly prognostic. Serial ctDNA patterns were also associated with recurrence risk. However, only 19 patients were evaluable, and the wide confidence interval and single-center design indicate substantial uncertainty. These findings should therefore be considered hypothesis-generating and require validation in larger prospective multicenter studies before ctDNA can guide treatment decisions in oligometastatic CRC. It also should not be used alone to select, escalate, or de-escalate treatment outside a prospective clinical study [146].

More generally, the clinical value of liquid biopsy in CRC depends on both the assay used and the clinical setting. As discussed by Malla et al., different ctDNA platforms have distinct strengths and weaknesses depending on the intended use, while Parikh et al. note that tumor-informed approaches may better distinguish tumor-derived alterations from non-tumor signals, such as clonal hematopoiesis [147]. Tumor-informed assays require sequencing of the resected tumor and subsequently track patient-specific variants in plasma [139,148]. They generally provide high analytical specificity but require available tumor tissue [139]. Tumor-naïve or plasma-only assays infer tumor-derived signals directly from plasma genomic and methylation patterns without prior tumor sequencing [145,147]. They are logistically simpler and can be used when tissue is unavailable, but their sensitivity may be lower at very low ctDNA fractions, and they require rigorous validation to distinguish tumor-derived signals from clonal hematopoiesis [139,145]. Consequently, results from different MRD platforms should not be considered interchangeable. Another important issue is the timing of blood collection, because testing performed too early after surgery may reduce sensitivity due to the higher levels of postoperative cfDNA. This helps explain why the performance of particular liquid-biopsy markers differs across settings, from the high specificity but limited sensitivity of mSEPT9 in screening to the stronger value of serial ctDNA measurements in MRD assessment [117,142,146,147]. Table 1 shows an overview of the main biomarker classes, their clinical applications, and current evidence and guideline status.

Blood-based screening has progressed from methylated SEPT9 testing [142] to cell-free DNA assays [143] and multi-cancer early detection (MCED) tests [149]. In the prospective ECLIPSE study, a cfDNA blood test detected 83.1% of colorectal cancers but only 13.2% of advanced precancerous lesions, indicating that blood-based testing may improve screening participation but cannot replace colonoscopy or FIT for prevention of precancerous lesions [143]. MCED assays such as PATHFINDER 2 are promising for detecting cancer signals from multiple organs, but their effect on CRC-specific mortality and overdiagnosis remains unproven [149].

Beyond screening, prospective ctDNA-guided management has expanded beyond the DYNAMIC trial [139]. NRG-GI005/COBRA evaluated a plasma-only, tumor-naïve assay in low-risk stage II colon cancer [150], whereas the CIRCULATE-Japan platform includes the observational GALAXY registry and the randomized phase III VEGA and ALTAIR trials [145,151]. VEGA evaluates treatment de-escalation in ctDNA-negative patients, while ALTAIR evaluates treatment escalation at molecular relapse in ctDNA-positive patients [151,152]. The UK TRACC program evaluates plasma-only ctDNA-guided adjuvant treatment [153,154], whereas BESPOKE is a prospective observational study based on a tumor-informed assay [148]. These studies address different clinical questions and should not be considered interchangeable (Table 2).

### 3.2. Multi-Omics and Spatial Profiling

Traditional genomic approaches do not fully describe the complexity of CRC, as DNA changes do not always translate into changes in protein expression. In response, multi-layered strategies involving genomics, transcriptomics, proteomics, and phosphoproteomics have been developed. Guo et al. performed whole-exome, transcriptomic, proteomic, and phosphoproteomic analyses on paired tumor and normal-adjacent samples from 144 CRC patients and identified three molecular subtypes associated with differences in prognosis and TME. Importantly, the authors emphasize that the CMS classification remains useful, but does not fully reflect CRC heterogeneity because it is based on mRNA data alone. In this setting, multi-omics can complement CMS by adding proteomic and phosphoproteomic information and by identifying biologically distinct groups, including the S_I subtype, which was associated with the worst prognosis, a higher proportion of early-onset CRC, and a higher stromal score [157]. However, the S_I subtype still requires replication in independent external cohorts and has not been incorporated into routine CRC molecular classification systems [157]. From this perspective, multi-omics is not only a broader descriptive approach but also a way to link clinically relevant tumor behavior to layers of biology not captured by DNA alterations alone. In the study by Guo et al., the integration of genomic, transcriptomic, proteomic, and phosphoproteomic data refined molecular stratification and showed that differences between tumors are related not only to mutation patterns but also to the state of signaling pathways and the TME. In turn, Wu et al. describe single-cell multi-omics and spatial methods as approaches that make it possible to study biomolecules in their tissue context and to better capture biological heterogeneity. Guo et al. likewise note that single-cell and spatial transcriptomic approaches may further improve the characterization of cellular composition and spatial organization in CRC. Taken together, these data suggest that future biomarker models may combine longitudinal blood-based MRD assessment with tissue-based molecular classification and spatially resolved tumor features [157,158].

Building on these approaches, integrated biomarker models combine genomic, epigenetic, immune, and clinical features to characterize CRC heterogeneity, treatment response, and resistance [159]. However, multi-omics integration does not by itself establish clinical utility; such models require external validation, calibration, reproducibility, and demonstration of benefit beyond established clinicopathological and molecular factors [155,156,159]. Individual findings involving PTEN/EGFR, ARID1A, chemokine pathways, or aspirin response should therefore be treated as component-level or hypothesis-generating evidence rather than as validated integrated biomarkers [160,161,162,163].

The quickly growing amount of biomedical data is too much for traditional analysis methods. Because of this, more focus is going to artificial intelligence (AI) and machine learning (ML) techniques. These approaches can analyze large genomic and clinical datasets and identify patterns that may remain undetectable using conventional analytical methods [155,156].

## 4. Clinical Applications

The previous sections described the biological basis and diagnostic relevance of individual CRC biomarkers. This section focuses on how clinicians use these biomarkers in decision-making, including treatment selection, resistance monitoring, and longitudinal disease assessment. Treatment selection for metastatic or recurrent disease is increasingly guided by molecular profiling. According to the ESMO and NCCN guidelines, first-line therapy depends on molecular status and tumor location [15,114,115]. In left-sided, RAS (KRAS/NRAS) and BRAF wild-type, MSS adenocarcinomas, the recommended approach combines EGFR inhibitors (cetuximab or panitumumab) with chemotherapy. For previously untreated BRAF V600E–mutated mCRC, encorafenib plus cetuximab combined with mFOLFOX6 is supported by the BREAKWATER trial [164], whereas the encorafenib–cetuximab doublet is an established option for previously treated mCRC based on the BEACON study [90]. Tumors harboring KRAS or NRAS mutations are generally treated with chemotherapy combined with an antiangiogenic agent such as bevacizumab in the first-line setting, whereas aflibercept plus FOLFIRI is an established second-line option after prior oxaliplatin-based therapy [165]. In later lines, targeted therapies include cetuximab with adagrasib for KRAS G12C, trastuzumab-based regimens for HER2-positive disease, and larotrectinib for NTRK fusions [15,114,115,166].

Biomarker-related heterogeneity also affects antiangiogenic therapy. c-MET overexpression has been associated with shorter overall and progression-free survival during bevacizumab treatment, whereas high MCL-1 expression has been associated with more favorable outcomes; however, neither marker is sufficiently validated for routine treatment selection [60,167]. Their potential role in resistance to antiangiogenic therapy remains investigational and requires prospective validation.

### Anti-EGFR Therapy: Predictive Biomarkers and Resistance Mechanisms

Anti-EGFR monoclonal antibodies (mAbs) such as cetuximab and panitumumab show benefit in RAS wild-type mCRC, yet responses vary markedly. Several biomarkers refine patient selection. High EGFR gene copy number (GCN ≥ 4) and increased EGFR expression correlate with improved clinical benefit, PFS, and OS [168,169]. In addition to EGFR copy number and expression, researchers are also looking at other possible markers such as ligand and co-receptor activity, epigenetic changes, circulating RNA profiles, and telomere-related features. These markers have been associated with anti-EGFR response mainly in exploratory translational or retrospective studies. RAS status remains the principal guideline-supported biomarker for anti-EGFR treatment selection, whereas EGFR gene copy number, EGFR expression, and the other candidate markers described above are not validated standalone criteria for routine treatment selection [15,115]. However, assay methods, thresholds, patient populations, and clinical endpoints vary substantially. These biomarkers are therefore not currently sufficient for routine treatment selection and should be regarded as hypothesis-generating. Prospective multicenter validation using standardized assays and clinically relevant endpoints is required before they can be incorporated into treatment guidelines [118,121,122,170,171,172,173,174,175,176,177].

Resistance to anti-EGFR therapy can be grouped into three interacting categories. First, target-gene alterations include acquired mutations in KRAS, NRAS, and BRAF, as well as mutations in the EGFR extracellular domain that may reactivate downstream signaling or impair antibody binding [178,179]. Second, compensatory pathway activation involves HER2/ERBB2, PIK3CA/PTEN dysregulation, and parallel PI3K/AKT or MAPK signaling, allowing tumor cells to maintain proliferative signaling despite EGFR blockade [81,180]. Third, tumor-microenvironment-mediated resistance may involve amphiregulin and TGF-α signaling, angiogenic pathways, stromal interactions, and immune-contexture effects [160,181,182]. Most of these mechanisms remain investigational and should not be used as standalone biomarkers for routine treatment selection. Serial ctDNA may help detect emerging resistance alterations, but its results should be interpreted together with clinical and radiological findings [160,181,183].

## 5. Future Perspectives

### 5.1. From Single Biomarkers to Integrated Molecular Profiles

CRC biomarker research is moving from isolated single-marker analyses toward integrated molecular profiles that combine genomic, transcriptomic, epigenetic, proteomic, spatial, and clinical information [159]. The priority is to demonstrate reproducible clinical value and benefit beyond established clinicopathological and molecular factors through external validation and prospective assessment of patient outcomes. Key questions include which biomarker combinations provide clinically meaningful information despite established markers, where they are most useful, and whether implementing them improves patient outcomes.

### 5.2. Molecular Tumor Evolution and Adaptive Treatment Strategies

Molecular tumor evolution creates a rationale for adaptive biomarker-guided treatment, but detecting a molecular change does not by itself prove that changing therapy improves outcomes [145,184]. Adaptive strategies should therefore be evaluated in prospective interventional studies with predefined sampling intervals, actionable thresholds, clinically relevant endpoints, and assessment of patient benefit [140,184].

### 5.3. Challenges in Clinical Translation and Standardization of Advanced CRC Biomarkers

Clinical translation requires standardized preanalytical and analytical procedures across ctDNA, circulating tumor cell, microRNA, methylation, and tissue-based assays, as well as assay-specific thresholds, quality control, and evidence that results are comparable across platforms [7,126,140]. Preanalytical variables matter equally: the interval between surgery and blood collection, postoperative cfDNA release, and the timing of serial blood draws can materially affect ctDNA readouts. Therefore, postoperative sampling windows should be predefined and harmonized across studies before ctDNA testing can be widely adopted [140]. Intratumoral heterogeneity, including variation in EGFR gene copy number, further complicates biomarker interpretation [168]. Regulatory approval, guideline inclusion, analytical performance, and prognostic association should be considered separate levels of evidence [140]. For ctDNA-based approaches, implementation also depends on the clinical setting, tissue availability, assay design, and the clinical action triggered by the result [140,148]. Prospective studies must demonstrate that biomarker-guided decisions improve patient outcomes and are clinically and economically justified before routine implementation [141,185]. For example, in the randomized phase 2/3 DYNAMIC-III trial in stage III colon cancer, ctDNA-guided de-escalation reduced oxaliplatin exposure in ctDNA-negative patients but did not meet the non-inferiority margin for RFS, whereas treatment escalation in ctDNA-positive patients did not improve RFS [186].

### 5.4. Future Directions

Future research should prioritize clinically actionable, scenario-specific biomarker strategies rather than discovery-only panels. Some of the most important questions include which biomarker combinations add value beyond established clinicopathological and molecular factors, how to integrate spatial and longitudinal information, and whether AI-supported models improve clinical decision-making and patient outcomes. Single-cell, spatial, liquid-biopsy, and AI-based approaches should therefore be evaluated in externally validated prospective studies with predefined clinical endpoints and transparent reporting [155,156,157,158]. Biomarkers should complement, rather than replace, established pathology, imaging, and clinical assessment.

## 6. Conclusions

Conventional biomarkers, including MSI/dMMR, RAS, BRAF, HER2, and CEA, remain the cornerstone of current CRC clinical practice because they provide the most established basis for treatment selection, surveillance, and prognostic assessment. Liquid biopsy, particularly ctDNA, offers substantial promise for minimal residual disease detection, recurrence monitoring, and longitudinal assessment of tumor evolution. However, assay heterogeneity, preanalytical variability, tumor shedding, clonal hematopoiesis, and the need to demonstrate benefit from ctDNA-guided interventions continue to limit routine implementation. Multi-omics, spatial transcriptomics, and artificial-intelligence-driven approaches represent important future directions for characterizing tumor heterogeneity and treatment response. Nevertheless, these technologies require harmonized methods, external validation, and large-scale prospective studies demonstrating improved patient outcomes before routine clinical deployment. Biomarkers should therefore complement, rather than replace, established pathology, imaging, and clinical assessment.

## Figures and Tables

**Table 1:** Comparison of biomarker approaches discussed in this section ^a^ [117,142,146,147].

Marker	Clinical Use	Evidence and Guideline Status	Limitations
ctDNA mutation analysis	MRD assessment, recurrence-risk stratification, and monitoring after radical treatment.	Prospective randomized evidence supports ctDNA-guided decisions in selected stage II settings, but broader implementation remains assay- and scenario-specific [139].	Performance depends on disease setting, metastatic site, assay, and timing of blood collection; testing too early after surgery may lower sensitivity.
cfDNA methylation markers (e.g., mSEPT9)	Blood-based detection/screening and epigenetic characterization of CRC.	Prospective screening evidence is available, but sensitivity for advanced precancerous lesions is limited; these tests do not replace FIT or colonoscopy [142].	mSEPT9 has high specificity but limited sensitivity in asymptomatic CRC and is therefore insufficient as a standalone screening marker.
Circulating miRNA/lncRNA	Diagnostic and prognostic support; possible reflection of aggressiveness, EMT, and treatment response.	Mainly exploratory, retrospective, or single-study evidence; no standardized clinical assay or routine guideline recommendation [126].	Biologically promising, but still not clinically standardized in the cited literature.
Integrated plasma assays (mutation + methylation; e.g., Guardant Reveal)	Tumor-naïve MRD detection without tumor sequencing and post-treatment risk stratification.	Prospective observational, protocol, and clinical-trial evidence for MRD assessment; platforms should not be considered interchangeable [145,147].	Useful when tissue is unavailable and logistically simpler, but results remain assay-specific and evidence in oligometastatic CRC comes from small cohorts.
Tissue multi-omics/spatial transcriptomics	Molecular subtyping, microenvironment characterization, and heterogeneity mapping.	Research-stage evidence; no established routine regulatory or guideline status [27,28,29].	Biologically informative, but tissue-based and not yet clinically standardized; additional single-cell/spatial studies and clinical validation are needed.

^a^ Most plasma-based assays lack harmonized preanalytical and analytical detection platforms. Therefore, results delivered by different commercial kits or assay versions cannot be directly compared without assay-specific validation [117].

**Table 2:** Clinical scenarios, biomarkers, evidence level, and guideline status [27,28,29,139,142,145,148].

Clinical Scenario	Biomarker or Test	Evidence Level	Current Clinical or Guideline Status
CRC screening	Colonoscopy and FIT	Established prospective evidence and guideline-supported screening practice [13]	Guideline-supported standard screening methods
CRC screening	Methylated SEPT9, cfDNA blood tests, and MCED assays	Prospective evidence is available, but sensitivity for advanced precancerous lesions remains limited. MCED outcome benefit is not yet established [142,143,149]	May complement screening strategies, but should not replace colonoscopy or FIT [143,149]
Postoperative MRD in localized CRC	Tumor-informed ctDNA assays	Prospective randomized evidence in selected stage II settings [139]	Clinically informative, but treatment-guiding implementation remains setting- and assay-specific
Postoperative MRD without tumor sequencing	Tumor-naïve or plasma-only ctDNA assays	Prospective observational, protocol, and clinical-trial evidence [145,147]	Not interchangeable with tumor-informed assays; broader routine use requires further validation
Advanced CRC treatment selection	MSI/MMR, RAS, BRAF, and HER2 status	Clinical-trial and guideline-supported evidence [11,15,55,63,103]	Clinically actionable biomarkers used in routine treatment selection [15,34,114,115]
Exploratory biomarker development	miRNA, lncRNA, SNPs, telomere length, and chromosomal alterations	Mainly retrospective, single-study, or exploratory evidence [122,125,126,127]	Investigational; no standardized assay or routine guideline recommendation [119,121,122,125]
Integrated biomarker models	Multi-omics, spatial profiling, and AI-based models	Research-stage evidence; external validation and clinical utility remain limited [27,28,29,155,156]	Not routinely guideline-supported; prospective validation is required

## Data Availability

No new data were created or analyzed in this study. Data sharing is not applicable to this article.
